# Application of Omics Analysis in the Clinical Practice and Research of Transthyretin Amyloidosis

**DOI:** 10.3390/genes17030333

**Published:** 2026-03-18

**Authors:** Hidenori Moriyama, Faiyza Akil Shaikh, Toshifumi Yokota

**Affiliations:** 1 Department of Medical Genetics, Faculty of Medicine and Dentistry, University of Alberta, Edmonton, AB T6G 2H7, Canada; 2 The Friends of Garrett Cumming Research & Muscular Dystrophy Canada, HM Toupin Neurological Sciences Research, Edmonton, AB T6G 2H7, Canada

**Keywords:** transthyretin amyloidosis, transthyretin, omics, genomics, proteomics, biomarker, cardiomyopathy, neuropathy

## Abstract

Transthyretin amyloidosis (ATTR) is a progressive disease characterized by systemic deposition of transthyretin-derived amyloid. Although the recent advent of disease-modifying therapies has expanded treatment options, substantial unmet needs remain, such as understanding disease heterogeneity, predicting treatment response, and prognostic stratification. In this review, we highlight the current and emerging roles of omics technologies in both clinical and research settings of ATTR, including genomics and its integration with other modalities. Currently, omics technologies are applied in clinical settings for accurate disease typing. Clinical samples are utilized to identify risk factors beyond specific transthyretin variants via genomics and epigenomics and to discover promising biomarkers via proteomics. Accumulating findings from omics analyses using cell and animal models are also facilitating the elucidation of the complex pathology of ATTR. Nevertheless, the application of omics analysis in ATTR research is still developing. Moving forward, it is expected to play a central role in accumulating datasets, leveraging cutting-edge technologies, utilizing integrated multi-omics, and bridging basic and clinical research. These advancements are expected to further accelerate the implementation of next-generation therapeutic strategies and precision medicine.

## 1. Introduction

### 1.1. Overview of Transthyretin Amyloidosis

Amyloidosis is a collective term for a group of diseases in which fibrous proteins rich in β-sheet structures, known as amyloid, deposit in various organs throughout the body, causing organ dysfunction. It is known that over 30 types of precursor proteins can undergo protein misfolding to form amyloid. Among these, transthyretin (TTR) amyloidosis (ATTR) is a clinically significant disease alongside light-chain amyloidosis (AL) caused by immunoglobulin light chains [1,2,3]. TTR is primarily produced in the liver and circulates in the blood as a tetramer, functioning to transport thyroxine and retinol-binding protein [4]. It is hypothesized that when the stability of this tetrameric structure is compromised, it dissociates into monomers, undergoes misfolding, and ultimately forms insoluble TTR amyloid fibrils that deposit in various tissues [5,6].

ATTR is broadly classified based on its etiology into wild-type ATTR (ATTRwt) and hereditary ATTR (ATTRv). ATTRwt is caused by deposition of wild-type transthyretin in the absence of pathogenic *TTR* variants, likely triggered by age-related breakdown of protein homeostasis, and predominantly affects elderly men [7,8]. In contrast, ATTRv follows an autosomal dominant inheritance pattern and is caused by pathogenic variants in the *TTR* gene; to date, more than 130 disease-associated TTR variants have been reported. The age of onset, clinical symptoms, severity, and prognosis of the disease vary widely [3,9]. In ATTRwt, TTR deposition occurs systemically across various organs but mainly affects joints and ligaments—causing carpal tunnel syndrome and spinal stenosis—and the heart, leading to cardiac hypertrophy, arrhythmia, and heart failure [2,3,10,11]. Bilateral carpal tunnel syndrome is one of the symptoms appearing in the early stages of the disease and generally precedes cardiac lesions [12]. Cardiac involvement contributes significantly to mortality and morbidity, resulting in cardiac dysfunction, heart failure, repeated hospitalizations, and ultimately death [7]. In clinical practice, it is an important differential diagnosis for heart failure with preserved ejection fraction (HFpEF) and left ventricular hypertrophy, and it has been revealed that comorbidity with aortic stenosis is frequent [13,14,15]. The clinical presentation of ATTRv is diverse, with polyneuropathy and cardiomyopathy being the typical phenotypes; depending on the variant, one may predominate, or both may coexist. Symptoms of polyneuropathy include sensory blunting, numbness, and pain, as well as orthostatic hypotension and gastrointestinal symptoms due to autonomic dysfunction [16,17]. Historically, definitive diagnosis requires the demonstration of amyloid deposition via tissue biopsy and the identification of the precursor protein through immunohistochemistry or mass spectrometry. However, at present, an increasing number of cases are being diagnosed at an earlier stage through non-invasive approaches. Echocardiography, cardiac CT, and cardiac MRI serve as useful screening tools for the differential diagnosis of cardiomyopathies. Bone scintigraphy, using Tc-99m PYP, DPD, or HMDP, is central to the non-invasive diagnosis of ATTR cardiomyopathy (ATTR-CM), and a definitive diagnosis can be established by the presence of Grade 2 or 3 cardiac uptake on bone scintigraphy in the absence of monoclonal protein [18].

The therapeutic strategy for ATTR has undergone a dramatic transformation over the last decade, shifting from an era centered on symptomatic treatment to the establishment of disease-modifying therapies that intervene in the fundamental process of amyloid formation [7,11,19,20]. Currently, clinical application is centered on two main approaches. The first involves TTR stabilizers that inhibit the dissociation of TTR tetramers. One such stabilizer is Tafadimis, which has established efficacy in improving long-term prognosis [21]. In addition to this, the next-generation drug Acoramidis, which provides more potent structural stabilization, has achieved favorable results in a Phase 3 ATTRibute-CM trial (Efficacy and Safety of AG10 in Subjects With Transthyretin Amyloid Cardiomyopathy; NCT03860935) [22]. The second approach involves TTR production inhibitors that inhibit TTR synthesis in the liver using RNA interference or antisense oligonucleotides. In this field, Vutrisiran and Eplontersen, which offer more convenient administration, have emerged. Notably, in the current Phase 3 HELIOS-B trial (A Study to Evaluate Vutrisiran in Patients With Transthyretin Amyloidosis With Cardiomyopathy; NCT04153149), Vutrisiran was associated with reduced all-cause mortality and a lower incidence of cardiovascular events in patients with cardiomyopathy [23]. These findings suggest that its indication could be expanded from neuropathy to include cardiac involvement. Furthermore, a paradigm shift is underway, moving beyond merely suppressing disease progression toward more curative approaches. NTLA-2001, an in vivo gene-editing therapeutic based on CRISPR–Cas9 technology, is being developed with the goal of “One-and-done” treatment via permanent TTR knockdown through a single dose, raising hopes for liberation from the burden of lifelong treatment [24,25]. Additionally, development is proceeding on anti-amyloid antibodies such as NI006 aimed at removing amyloid already deposited in tissues and restoring organ function [26]. Going forward, along with the clinical application of these novel modalities, the establishment of combination therapies using drugs with different mechanisms of action and optimal treatment sequences according to disease stage are becoming critical challenges.

### 1.2. Omics Approaches

As described above, while the management of ATTR has achieved dramatic progress with the advent of disease-modifying therapies, new challenges have surfaced, such as understanding disease heterogeneity, predicting treatment response, and prognostic stratification [7,27]. To address these clinical issues and elucidate the complex pathological mechanisms at the molecular level—mechanisms that extend beyond mere “amyloid deposition in organs”—it is essential to utilize omics analysis to enable comprehensive data acquisition, rather than relying solely on conventional fragmentary analyses. Omics is a field of study that involves the comprehensive analysis of molecular information within living organisms, such as genes, proteins, and metabolites. Unlike conventional methods that focus on specific individual molecules, omics aims to elucidate a holistic view of biological functions and disease mechanisms by analyzing vast amounts of biological data simultaneously, and is expected to contribute to the realization of personalized medicine. Depending on the molecules being analyzed, this approach includes genomics, epigenomics, transcriptomics, proteomics, and metabolomics. In recent years, multi-omics analysis, which integrates and analyzes multiple types of omics data, has also become increasingly prevalent [28,29]. In Alzheimer’s disease, another representative amyloid-related disorder, the application of omics is advancing, leading to the identification of risk genes other than *APOE* and processes related to disease onset [30,31,32]. Conversely, the use of omics in ATTR is still developing. While several pilot studies have emerged, the number of reports—particularly those involving large-scale cohorts—remains limited. In this review, we outline current findings regarding the use of omics in the diagnosis, elucidation of pathology, and biomarker exploration of ATTR amyloidosis, and discuss future perspectives for ATTR research utilizing these technologies.

## 2. Application of Proteomics for Amyloid Typing in Clinical Practice

**Figure 1:**
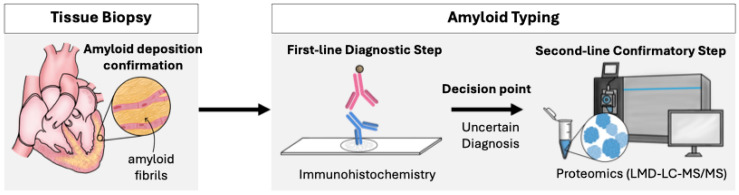
Role of proteomics in the diagnosis of amyloidosis. When amyloid deposition is confirmed in biopsy specimens, but definitive amyloid typing cannot be achieved by immunohistochemistry, proteomic analysis is useful for identifying the amyloid precursor protein. LMD, laser microdissection; LC-MS/MS, liquid chromatography–tandem mass spectrometry.

In clinical practice, accurate determination of the amyloid type, referred to as amyloid typing, is essential for establishing subsequent disease management strategies. The natural history, organs involved, and associated complications differ depending on the precursor protein, and therapeutic paradigms therefore differ fundamentally. For example, AL is treated with chemotherapy targeting clonal plasma cells, whereas ATTR is managed with TTR stabilizers or TTR synthesis–inhibiting therapies. Furthermore, when an amyloid type with a genetic contribution is identified, this has important implications for genetic counseling and screening of family members. Proteomics is utilized for amyloid typing and is incorporated into the diagnostic workflow [2,33]. Although bone scintigraphy is a useful non-invasive tool for ATTR, tissue biopsy is required when definitive diagnosis cannot be obtained. While amyloid deposition is confirmed by biopsy and amyloid typing is attempted via immunohistochemistry, diagnosis is often difficult in a significant number of cases due to limitations in antibody sensitivity and specificity. Implementing proteomics on collected samples at this stage significantly improves sensitivity and specificity, enabling the unbiased and comprehensive identification of amyloid precursor proteins [33,34,35,36] (Figure 1). The method of performing proteomics after harvesting amyloid deposition areas using laser microdissection has become standard practice [34,37].

The following case demonstrates the clinical utility of proteomics combined with laser microdissection in overcoming diagnostic ambiguity in amyloid typing. The patient presented with a complex clinical picture characterized by strongly positive bone scintigraphy findings suggestive of ATTR-CM, alongside nephrotic syndrome and monoclonal gammopathy raising suspicion for AL. This coexistence made clinical diagnosis particularly challenging. Therefore, an endomyocardial biopsy was performed to establish a definitive diagnosis. Although amyloid deposition was confirmed in the biopsy specimen, immunohistochemical analysis revealed positivity for both TTR and immunoglobulin light chain in different regions within the same sample, rendering pathological typing inconclusive. Given that the concurrent presence of ATTR and AL is extremely rare and generally not anticipated, further investigation was warranted. Accordingly, the respective amyloid-positive regions were selectively isolated using laser microdissection, followed by separate proteomic analyses. These analyses independently identified TTR protein in one region and immunoglobulin light chain protein in another, leading to a definitive diagnosis of the exceptionally rare condition of concurrent ATTR and AL [38]. Importantly, the accurate identification of this coexistence extended beyond mere disease classification. In this case, the patient was determined to have a combination of relatively slowly progressive ATTR and advanced-stage AL, which is associated with a poor prognosis. This precise diagnosis enabled appropriate and transparent discussions with the patient and family regarding the potential for rapid clinical deterioration, anticipated complications, and unfavorable prognosis, thereby facilitating informed decision-making with respect to optimal therapeutic strategies and end-of-life care planning.

Proteomics of biopsy samples also enables the detection of rare amyloid types that were difficult to detect with immunohistochemistry. A comprehensive review of 16,175 specimens derived from various organs analyzed by shotgun proteomics at the Mayo Clinic identified numerous rare amyloid types and presented a “type-by-organ map” demonstrating the organ tropism of 18 rare types [33]. Furthermore, the same study demonstrated that integration with bioinformatics approaches enables the identification of pathogenic protein variants in hereditary amyloidosis.

In summary, the clinical integration of proteomic analysis provides a decisive diagnostic foundation with high objectivity and sensitivity, even for challenging cases—including rare amyloid types—that are difficult to characterize using conventional immunohistochemistry. Ultimately, omics analysis serves as an indispensable bridge in contemporary amyloidosis management, linking “precision diagnosis” directly to “optimal therapeutic intervention and patient care.”

## 3. Research Applications of Omics in Patient Sample Analysis

Omics research using patient samples is considered applicable to a wide range of purposes, from biomarker discovery to the elucidation of the pathogenesis of ATTR. In this section, we review research examples utilizing various omics technologies and discuss future challenges in the field.

### 3.1. Serum Biomarker Discovery

The discovery of serum biomarkers in ATTR is a critical theme addressing multifaceted clinical and scientific needs, including non-invasive diagnosis, early diagnosis and screening, prediction of disease onset and progression, monitoring of therapeutic efficacy, promotion of precision medicine, and the identification of novel therapeutic targets.

#### 3.1.1. Proteomic Evidence for Heterogeneity in ATTR

In early studies performing proteomics using serum from 8 patients with ATTR-CM, 8 patients with ATTR-polyneuropathy, and 10 controls, proteins specific to ATTR-CM, including low levels of TTR, were identified [39]. Similarly, a study comparing the serum profiles of 8 ATTRv patients, 10 ATTRwt patients, and 10 healthy controls revealed that serum protein profiles differ between the two disease types despite both being ATTR. In particular, the ATTRv group showed specific changes in 14 proteins, including retinol-binding protein 4 (RBP4), while the ATTRwt group exhibited specific variations in 27 proteins, including apolipoprotein E and complement component C7 [40]. Despite the limited sample sizes in these pilot studies, proteomic analysis captured distinct serum protein profiles associated with specific organ involvement and the presence of *TTR* variants. These findings highlight the significant molecular heterogeneity inherent in ATTR, even among cases sharing the same precursor protein. As a next step, validating these findings in larger cohorts to ensure reproducibility is expected to facilitate the clinical application of serum protein profiling for supplemental diagnosis—enabling detailed patient stratification based on organ involvement and genetic status—as well as for precise therapeutic monitoring.

#### 3.1.2. Identification of Biomarkers for Disease Progression and Treatment Response in ATTRv

A prime example of biomarker discovery where omics analysis proved useful and led to practical application is the identification of neurofilament light chain (NfL) as a marker for neuronal damage in ATTRv. In a study utilizing serum samples collected during the Phase 3 APOLLO study (a randomized, double-blind, placebo-controlled trial of patisiran in patients with ATTRv with polyneuropathy; NCT01960348) [41], proteomics identified NfL as a valuable biomarker for ATTRv [42]. NfL is a protein that maintains the structure and shape of nerve cells and functions as a marker for neuronal axonal injury or degeneration. In this study, serum levels of over 1000 proteins were measured in 136 patisiran-treated ATTRv patients at baseline, 9 months, and 18 months, in addition to placebo-treated ATTRv patients and 57 healthy controls. Among the 66 proteins that showed significant differences in the patisiran group compared to the placebo group, NfL exhibited the most significant change. The proteome at 18 months in the patisiran treatment group tended to approach that of the healthy control group. Furthermore, longitudinally, NfL levels increased in the placebo group while demonstrating a decrease in the patisiran group. These findings suggested that NfL functions as a biomarker for nerve damage and polyneuropathy in ATTRv, potentially facilitating the monitoring of disease progression. The utility of NfL as a robust indicator of disease progression and treatment response has been confirmed in subsequent analyses of real-world clinical samples [43]. While NfL represents a breakthrough biomarker identified through proteomics, several caveats remain regarding its clinical application. First, since NfL is a non-specific marker reflecting axonal damage, its elevation does not provide direct evidence of amyloid deposition, nor is it pathognomonic for neuropathy specifically caused by ATTRv. In other words, NfL levels alone are insufficient for a definitive diagnosis of ATTR. Second, it is not yet established as a standalone early diagnostic marker; the extent of its sensitivity in predicting disease onset or contributing to early diagnosis remains to be fully elucidated.

Other studies are also underway to identify biomarkers for monitoring disease progression and the efficacy of disease-modifying therapies. Nugroho et al. conducted a comparative plasma analysis of healthy controls, asymptomatic V30M *TTR* variant carriers, untreated patients, and tafamidis-treated patients from a Portuguese cohort. They utilized differential precipitation of proteins—a technique for fractionating complex protein mixtures—combined with mass spectrometry-based proteomics and peptidomics [44]. Their analysis revealed evidence of inflammation, complement activation, and elevated levels of oxidative modifications on TTR and APOE, alongside increased protease activity characterized by enhanced peptide fragmentation in patients. Notably, tafamidis treatment was shown to ameliorate or normalize these aberrant proteolytic and oxidative profiles. These findings demonstrate that V30M ATTRv is not merely a protein aggregation disorder but a systemic pathology involving complex interplays between inflammation and oxidative stress. Future studies comparing these signatures with other ATTRv variants beyond V30M, as well as other systemic inflammatory diseases, and investigating their association with individual therapeutic responses will be essential for further elucidating the pathogenesis and realizing personalized medicine.

#### 3.1.3. Metabolomic Studies

Data regarding serum metabolomics in ATTRv patients have also been reported. A study utilizing GC-MS and LC-MS metabolomics on serum samples from 27 ATTRv patients with V30M *TTR* variant, asymptomatic V30M *TTR* carriers, and 26 controls demonstrated the existence of significantly different metabolic profiles in ATTRv patients, revealing decreased levels of multiple amino acids such as tryptophan and phenylalanine [45]. However, this study could not identify differences in metabolic profiles between controls and asymptomatic carriers. Another small-scale study comparatively analyzed the metabolic profiles of 26 types of serum amino acids and 10 types of free fatty acids in 12 ATTRv patients and 12 healthy controls. The results revealed significantly lower levels of palmitic acid—a major saturated fatty acid—in ATTRv patients compared to controls, suggesting that this metabolic alteration may reflect pathological processes such as mitochondrial dysfunction and neuroinflammation. In contrast, no significant differences were observed in the amino acid profiles [46]. The rationale for conducting metabolomics in addition to proteomics lies in its capacity to provide a comprehensive understanding of how protein-level alterations are ultimately reflected in the metabolic phenotype as a final biological response. As with many other studies, validation in larger cohorts is expected to contribute to a multi-dimensional elucidation of the pathogenesis and to capturing the specific characteristics of asymptomatic carriers. Ultimately, such efforts will facilitate earlier diagnosis and more precise patient stratification.

#### 3.1.4. ATTR as a Differential Diagnosis of Cardiomyopathy

The number of patients with ATTR-CM has increased rapidly in recent years, making the appropriate identification of ATTR-CM among patients presenting with left ventricular hypertrophy (LVH) a clinically important issue. Serum proteome analysis was performed on pre-treatment ATTR-CM patients, ATTR-CM patients after the induction of tafamidis treatment, and patients with myocardial hypertrophy in whom ATTR was excluded, in an attempt to identify potential biomarkers for ATTR-CM diagnosis and the evaluation of tafamidis treatment efficacy [47]. Characteristic protein changes were observed in each group; specifically, prior to the initiation of specific therapy, ATTR-CM patients showed elevated levels of multiple proteins, including ceruloplasmin, apolipoprotein E, SERPINA1, and cDNA FLJ54111, which is highly similar to serum transferrin. Notably, levels of SERPINA1 and cDNA FLJ54111 were found to decrease in the tafamidis treatment group. This study suggests that specific serum proteome profiles can differentiate ATTR-CM from other forms of myocardial hypertrophy, though further validation in larger external cohorts is warranted.

Furthermore, Akita et al. conducted a large-scale proteomic profiling using the SomaScan assay to analyze 4979 proteins in plasma samples from 1415 patients with LVH, including ATTR-CM. While this study primarily focused on hypertrophic cardiomyopathy (HCM)—demonstrating that a five-protein model could distinguish HCM with a high accuracy of 0.86 for the area under the curve—it also identified a distinct set of molecules significantly altered in ATTR-CM. Given the scale of this dataset, further investigations are expected to yield specific biomarkers that will enhance the diagnostic precision and mechanistic understanding of ATTR-CM [48].

### 3.2. Tissue Sample Analysis

In recent years, omics analysis utilizing clinical samples obtained via biopsy has been actively pursued, significantly contributing to the elucidation of disease pathology.

Amyloid deposition areas are characterized by the accumulation of not only precursor proteins but also specific proteins known as the “amyloid signature” [49,50]. Kourelis et al. conducted a comprehensive proteomic analysis of amyloid plaques within cardiac tissue samples from 292 ATTR-CM patients and 139 AL-CM patients to elucidate the specific proteome composition of cardiac amyloid plaques and its clinical significance [49]. The results revealed characteristic differences: ATTR plaques were rich in complement and contraction-related proteins, whereas AL plaques were rich in keratin. Furthermore, in a subset of ATTR patients, elevated levels of PIK3C3, an autophagy-related protein, were identified to be associated with reduced survival, suggesting the importance of autophagy pathways in the mechanism of cardiac tissue injury.

Netzel et al. performed proteomic analysis on whole endomyocardial biopsy samples from 76 ATTR patients and 27 AL patients [51]. They identified pathways common to both ATTR-CM and AL-CM, as well as pathways specific to ATTR-CM. In both ATTR-CM and AL-CM, extracellular matrix remodeling, fibrosis, epithelial–mesenchymal transition, and complement system activation were observed; these features were revealed to be associated with severe disease and poor prognosis. In ATTR-CM, particularly robust activation of the complement system was observed, suggesting the importance of immune-mediated reactions and antibody-related mechanisms. Based on these findings, the group reported that they have embarked on proof-of-concept research regarding complement inhibition for patients with advanced cardiac amyloidosis.

Omics analysis utilizing human tissue samples has significantly advanced our understanding of the microenvironment within amyloid-deposited organs, facilitating the development of novel therapies targeting affected tissues. However, since amyloid deposition is often heterogeneous throughout the organ, careful consideration must be given to sampling variability during data analysis and interpretation. Looking toward clinical application, biopsy samples are expected to evolve beyond simple amyloid typing to include disease stratification and the prediction of therapeutic responses. Nevertheless, given the invasive nature of biopsies and their associated time and cost constraints, the integration of these molecular insights with minimally invasive diagnostic approaches will be essential.

### 3.3. Genomic Analysis of Patients with Wild-Type Transthyretin Amyloidosis

As part of large-scale genomic analyses of ATTR, studies exploring *TTR* gene variants in general population cohorts have been reported [52,53]. Meanwhile, ATTRwt is defined as ATTR occurring in the absence of pathogenic *TTR* variants. Efforts have begun to elucidate the complex genetic background and regulatory mechanisms involved in its pathogenesis. Recently, studies utilizing genomics to identify risk factors other than the *TTR* gene have started to emerge.

In a report conducting genetic testing covering over 50 genes using multiple panels on familial cases from two ATTRwt families, no clear genetic variants were identified within the scope verified [54]. On the other hand, Moreno-Gázquez et al. performed targeted sequencing of 84 coding genes suspected of disease involvement using samples from 27 ATTRwt patients. Their analysis classified 17 variants across 14 genes—including those related to proteolysis such as *F2*, *PLAT*, *PSEN2*, extracellular chaperones such as *FGA*, *FGB*, and genes associated with other amyloid diseases such as *ABCA1*, *APP*, *GC*—as Variants of Uncertain Significance (VUS) [55]. While these findings suggest that genomic variants other than those in the *TTR* gene might influence ATTRwt, it remains unclear whether the frequency of these variants exceeds what would be expected by chance, as the study did not include a healthy control group.

Looking ahead, the utilization of more extensive genomic data—derived from genome-wide association studies (GWAS), whole-exome sequencing, and whole-genome sequencing—is expected to further accelerate the identification of risk factors and advance research into the polygenic risk and somatic variants associated with ATTRwt. A particularly noteworthy initiative in this field is the TTR GWAS, an international collaborative project [56]. This project is currently establishing a large-scale cohort involving thousands of ATTRv and ATTRwt cases, which is poised to become an invaluable data resource for future research. Furthermore, investigating non-coding regions, such as the TTR promoter and enhancer regions, as well as advancements in epigenetics, will likely provide critical insights into the clinical heterogeneity of ATTRwt amyloidosis—specifically regarding the marked variations in age of onset, organ involvement, progression rate, and treatment response.

### 3.4. Epigenomic Analysis of Patients with Hereditary Transthyretin Amyloidosis

ATTRv is defined as ATTR caused by pathogenic variants in the *TTR* gene; however, it is well recognized that there is marked interindividual variability in age at onset, penetrance, and clinical manifestations, even among patients carrying the same pathogenic variant. For example, the V30M *TTR* variant is associated with early-onset disease and high penetrance in Portuguese families, whereas in Swedish and non-endemic populations it typically presents with late-onset disease and low penetrance [57,58]. Such differences cannot be fully explained by the genetic variant alone, and epigenetic involvement has been proposed as one possible contributing factor. Indeed, epigenomics-based studies have demonstrated that, in ATTRv, epigenetic regulation, in addition to the underlying pathogenic variant, represents an important disease-modifying mechanism.

De Lillo et al. conducted an Epigenome-wide association study (EWAS) targeting 48 *TTR* variant carriers, including both symptomatic and asymptomatic individuals, and a control group of 32 non-carriers, performing a comprehensive investigation of over 700,000 DNA methylation sites [59]. Their findings revealed a significant reduction in methylation at specific sites within the *BACE2* gene, which is involved in amyloid metabolism, among variant carriers. Furthermore, they demonstrated that the V30M variant itself disrupts methylation sites within the *TTR* gene, resulting in hypomethylation. They also identified that the methylation status of the *B4GALT6* gene differs significantly between symptomatic and asymptomatic individuals, and pinpointed specific methylation sites associated with clinical symptoms such as cardiac involvement and carpal tunnel syndrome.

In another study, an EWAS was performed on 96 African Americans carrying the V122I variant to examine the relationship between methylation status and clinical symptoms [60]. Methylation changes associated with cardiac involvement were identified in genes such as *FAM129B*, *SKI*, and *GLS*, which are involved in critical biological processes, including amyloid transport and clearance, myocardial fibrosis, and regulation of muscle tissue.

These findings strongly suggest that epigenetic factors, in addition to genetic variants, play a pivotal role in determining the phenotype and age of onset in ATTRv. Future research should focus on validating these results in larger cohorts, performing functional analyses to discern whether the observed epigenetic alterations are a trigger for pathogenesis or a consequence of the disease, and exploring their potential application as clinical biomarkers.

## 4. Application of Omics in Preclinical Models

Although the in vitro reconstruction of TTR-derived amyloid fibrils that accurately replicate patient-derived structures remains challenging, research efforts are underway to elucidate the pathophysiology of ATTR by examining cellular structure, function, and responses following exposure to precursor proteins or amyloid fibrils.

To investigate the impact of amyloid fibrils on cardiac fibroblasts, Dittloff et al. cultured primary fibroblasts on glass substrates coated with TTR fibrils for 48 h and performed transcriptome analysis [61]. The results revealed a significant upregulation of 51 inflammation-related genes, specifically indicating the activation of IL-17, TNF, and NF-κB signaling pathways. Furthermore, proteomic-based cytokine screening showed that the secretion levels of several pro-inflammatory cytokines and chemokines increased more than fivefold compared to the control group. Although this study is limited by its short-term in vitro culture conditions, the findings suggest that amyloid deposition is not merely a physical obstruction but a pathological process that induces inflammation, consistent with results from human clinical samples [44,62]. These results also suggest that fibroblasts and specific inflammatory pathways may serve as promising novel therapeutic targets.

Qin et al. evaluated in vitro cellular responses to amyloid fibrils in the three major cardiac cell types: cardiomyocytes, endothelial cells, and fibroblasts—utilizing bulk RNA-sequencing to uncover comprehensive transcriptomic changes [63]. They generated human recombinant TTR proteins, including WT, V122I variant, and V30M variant, and their respective amyloid fibrils. The study demonstrated that exposure to TTR tetramers did not exhibit cytotoxicity. In contrast, upon exposure to TTR fibrils, cardiomyocytes exhibited cell death and the disruption of contractile structures, while endothelial cells showed gene expression changes associated with vascular remodeling alongside a significant decrease in survival rates. Conversely, fibroblasts demonstrated robust resistance while promoting collagen formation and the overproduction of extracellular matrix. These findings suggest that the cumulative effect of these distinct cell-type-specific responses contributes to the formation of the unique heart failure pathology observed in ATTR-CM.

Ghosh et al. investigated cellular responses to variant TTR proteins by exposing neuronal SH-SY5Y cells and AC16 cardiomyocytes to wild-type TTR as well as the V122I and L55P variant TTR proteins, followed by RNA sequencing and ATAC sequencing [64]. The study revealed that cellular responses vary depending on the specific TTR variant, suggesting a potential link to the diverse clinical profiles observed among different variants. Furthermore, treatment with tafamidis in the presence of variant TTR was found to suppress chromatin structural alterations and stress-response gene expression. Distinct gene expression patterns were observed between neuronal and cardiac cells; for instance, neuronal cells exhibited alterations in genes related to fatty acid metabolism and protein secretion, whereas cardiomyocytes showed an upregulation of gene sets associated with cardiac hypertrophy. Although previous reports indicate that TTR monomers and oligomers are cytotoxic [65,66], this study did not include steps to directly isolate and verify the denaturation or folding states of the protein. Consequently, the authors refrained from drawing definitive conclusions regarding which specific structural form—monomer, oligomer, or fibril—was responsible for the observed cellular responses.

Research utilizing human induced pluripotent stem cells to elucidate the role of the liver—the primary TTR-producing organ—in disease pathology has also been reported [67]. Single-cell RNA sequencing performed on hepatocyte-like cells expressing amyloidogenic TTR revealed that the production of variant TTR triggers the restructuring of intracellular proteostasis. Furthermore, the activation of the transcription factor ATF6 was found to induce chaperones such as BiP and PDIA4, resulting in the retention of variant TTR within the endoplasmic reticulum and a selective reduction in its secretion. These findings suggest the potential for a novel therapeutic strategy that prevents the release of toxic TTR by enhancing the protein quality control mechanisms of the liver.

These series of in vitro studies on cellular responses have elucidated part of the complex and heterogeneous pathological mechanisms of ATTR, forming a foundation for the exploration of novel therapeutic targets. As a next step, replication under physiological conditions is desired; however, in contrast to such cell-based analyses, accurately recapitulating ATTR in animal models has been historically considered difficult [68,69]. Consequently, there remain few reports on omics-based studies using in vivo models. Nevertheless, with the recent development of humanized models [70,71], it is expected that future omics analyses using in vivo models will yield novel insights.

## 5. Future Directions

**Figure 2:**
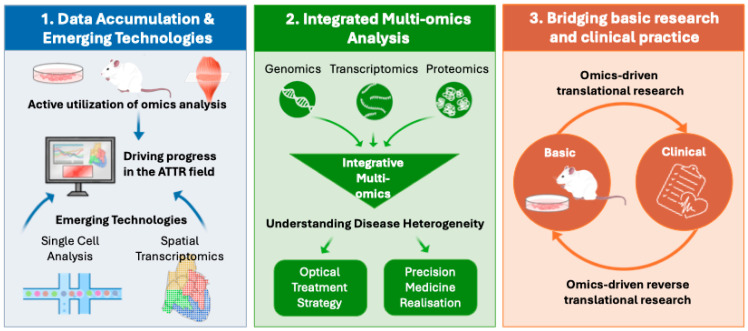
Future directions for omics-based research in transthyretin amyloidosis.

Table 1 summarizes the omics research examples discussed in the text. In the field of ATTR, omics-based approaches have already achieved tangible clinical impact, including their incorporation into diagnostic workflows for amyloid typing and their contribution to the identification of clinically relevant biomarkers such as NfL. At the same time, growing evidence indicates that ATTR-CM requires disease-specific therapeutic strategies distinct from conventional heart failure management [72,73], and that ATTR pathophysiology is considerably more complex than previously appreciated. These advances underscore the need for deeper molecular characterization of the disease. In this context, three key challenges emerge as important future directions for omics-based research in ATTR (Figure 2).

First, the systematic accumulation of omics datasets and the broader adoption of advanced analytical technologies are essential. To date, the number of omics studies and available datasets in ATTR remains limited. Expanded application of omics analyses across cellular models, animal models, and clinical samples is therefore critical to advancing the field as a whole. As discussed, many findings from clinical samples are derived from small patient cohorts with specific characteristics, such as affected organs or genetic variants. Consequently, further validation in larger external cohorts is required to determine the generalizability of these results. Promoting international large-scale projects, such as the TTR GWAS involving thousands of ATTR patients, will provide a clearer understanding of the overall landscape of this heterogeneous disease [56]. Moreover, emerging technologies such as single-cell and spatial transcriptomics—now widely applied in other disease areas—have scarcely been explored in ATTR. Applying these approaches to human biopsy specimens has the potential to elucidate microenvironmental changes surrounding amyloid deposits at high resolution, thereby uncovering previously unrecognized disease mechanisms and novel therapeutic targets.

Second, integrated multi-omics analyses are needed to define disease subtypes at the molecular level. A major future challenge lies in the integration of independently generated datasets, including genomic, transcriptomic, proteomic, and metabolomic data, to better capture disease heterogeneity [74,75]. Such integrative multi-omics approaches may enable molecular stratification of patients with differing rates of disease progression, organ tropism, and therapeutic responsiveness. This framework would provide a foundation for precision medicine in ATTR, facilitating the selection of optimal, individualized treatment strategies.

Third, omics analyses are expected to play a central role in bridging basic research and clinical practice. Indispensable to this goal are not only translational efforts—driving therapeutic development from cellular and animal models—but also reverse translational strategies that validate patient-derived hypotheses in experimental systems. In this reverse translational context, the rapid expansion of therapeutic options for ATTR offers a unique opportunity. Longitudinal omics profiling before and after treatment serves as a powerful approach to delineate how disease-modifying therapies reshape molecular networks at both tissue and systemic levels. Such analyses may ultimately lead to the identification of novel pharmacodynamic biomarkers and predictors of treatment response.

In summary, continued advances in omics-based research promise to propel our understanding of ATTR into a new era, accelerating the development of next-generation therapeutic strategies and the implementation of precision medicine for this complex disease.

## Figures and Tables

**Table 1:** Summary of Key Research Case Studies Utilizing Omics Technologies.

Category	Research Content (Sample and Analysis Method)	Ref.
**Pathogenesis**	Characterization of the proteome in amyloid-deposited hearts and comparison with AL-CM: Involvement of complement and autophagy-related proteins (Proteomics of cardiac biopsy samples)	[49,51]
**Pathogenesis**	Induction of inflammation by cardiac fibroblasts exposed to TTR fibrils (In vitro transcriptomics, proteomics)	[61]
**Pathogenesis**	Differences in cellular responses of cardiomyocytes, endothelial cells, and fibroblasts exposed to TTR fibrils (In vitro transcriptomics)	[63]
**Pathogenesis**	Differences in cellular responses of cardiomyocytes and neuronal cells to TTR variants (In vitro transcriptomics, epigenomics)	[64]
**Pathogenesis**	Restructuring of intracellular proteostasis in hepatocytes expressing variant TTR (Transcriptomics)	[67]
**Diagnosis**	Amyloid typing and detection of rare amyloids (Proteomics of biopsy samples)	[33,38]
**Classification**	Identification of amino acid and lipid changes in ATTRv patients (Serum metabolomics)	[45,46]
**Classification**	Characterization of the serum proteome in ATTR-CM patients among disease groups presenting with LVH (Serum proteomics)	[47,48]
**Classification**	Identification of epigenetic changes (DNA methylation) in variant carriers (Serum epigenomics)	[59,60]
**Classification**	Extraction of non-TTR genetic variants in ATTRwt (Serum genomics)	[55,56]
**Prognosis**	Identification of NfL as a nerve damage marker useful for assessing disease progression and treatment response in ATTRv (Serum proteomics)	[42]
**Prognosis**	Involvement of inflammation, oxidation, and complement system activation in ATTRv patients and changes following treatment initiation (Serum proteomics)	[44]

## Data Availability

No new data were created or analyzed in this study. Data sharing is not applicable to this article.
